# Retraction Note: Mir20a/106a-WTX axis regulates RhoGDIa/CDC42 signaling and colon cancer progression

**DOI:** 10.1038/s41467-023-41612-z

**Published:** 2023-09-18

**Authors:** Gui-fang Zhu, Yang-wei Xu, Jian Li, Hui-lin Niu, Wen-xia Ma, Jia Xu, Pei-rong Zhou, Xia Liu, Dan-li Ye, Xiao-rong Liu, Tao Yan, Wei-ke Zhai, Zhi-jun Xu, Chun Liu, Lei Wang, Hao Wang, Jia-mao Luo, Li Liu, Xuan-qi Li, Suiqun Guo, Hui-ping Jiang, Peng Shen, Hui-kuan Lin, Di-hua Yu, Yan-qing Ding, Qing-ling Zhang

**Affiliations:** 1 Department of Pathology, Nanfang Hospital, Southern Medical University, Guangzhou, GuangDong 510515 China; 2 Department of Pathology, School of Basic Medical Sciences, Southern Medical University, Guangzhou, GuangDong 510515 China; 3 Key Laboratory of Molecular Tumor Pathology of Guangdong Province, Guangzhou, GuangDong 510515 China; 4 Department of Oncological Sciences, Icahn School of Medicine at Mount Sinai, New York, New York, 10029 USA; 5 Nanfang Hospital/First clinical Medical School, Southern Medical University, Guangzhou, GuangDong 510515 China; 6 Hepatology Unit and Department of Infectious Diseases, Nanfang Hospital, Southern Medical University, Guangzhou, GuangDong 510515 China; 7 Department of Obstetrics and Gynecology, The Third Affiliated Hospital, Southern Medical University, Guangzhou, GuangDong 510630 China; 8 Department of Oncology, Nanfang Hospital, Southern Medical University, Guangzhou, GuangDong 510515 China; 9 Cancer Biology Comprehensive Cancer Center, Wake Forest Baptist Medical Center, Winston-Salem, NC 27157 USA; 10 Department of Molecular & Cellular Oncology, The University of Texas, MD Anderson Cancer Center, Houston, TX 77030 USA

Retraction to: *Nature Communications* 10.1038/s41467-018-07998-x published online 10 January 2019

The authors have retracted this article due to multiple imaging errors across several figures which call into the reliability of the data and the conclusions. In particular, we note there were errors in the colony formation images (Fig. 1j, Fig. 1l, 5j), migration images (Fig. 1i, 1h, 2h, 5h), western blots (Fig. 2e, Fig. 6a) and histology (Supplementary Fig. 4a). In addition, at the time of publication some experiments were lacking biological repeats (Fig 2e, 2h, 5h, 5j, 6a) We apologise for any inconvenience caused to the community. Qingling Zhang has stated on behalf of all authors that they agree to this retraction.

